# Longitudinal recovery patterns of penile length and the underexplored benefit of long-term phosphodiesterase-5 inhibitor use after radical prostatectomy

**DOI:** 10.1186/s12894-018-0341-8

**Published:** 2018-05-09

**Authors:** Young Suk Kwon, Nicholas Farber, Ji Woong Yu, Kevin Rhee, Christopher Han, Patrick Ney, Jeong Hee Hong, Paul Lee, Nikhil Gupta, Wun-Jae Kim, Isaac Yi Kim

**Affiliations:** 1 Section of Urologic Oncology, Rutgers Cancer Institute of New Jersey, 195 Little Albany Street, New Brunswick, NJ 08903 USA; 2 Division of Urology, Rutgers Robert Wood Johnson Medical School, New Brunswick, NJ USA; 3 Samsung Medical Center, Sungkyunkwan University School of Medicine, Seoul, Korea; 4 Department of Biostatistics, Rutgers School of Public Health, Piscataway, NJ USA; 5 Department of Urology, Dankook University College of Medicine, Chungnam, Korea; 6 Department of Urology, Chungbuk National University College of Medicine, Cheongju, Korea

**Keywords:** Penile length, PDE5 inhibitor, Radical prostatectomy, RP

## Abstract

**Background:**

Penile length (PL) shortening is an underreported phenomenon following radical prostatectomy (RP) and risk factors are not fully explored. We aimed to describe longitudinal patterns of PL recovery and evaluate factors predicting complete return to baseline PL.

**Methods:**

PL measurement was performed during a preoperative and postoperative follow-up visits at 7 days and 3, 6, 9, and 12 months. Patients who completely recovered (CR: *N* = 397) their preoperative stretched PL measured during at least one of their follow-up visits were compared to those with incomplete recovery (IR: *N* = 131). Recovery patterns were analyzed for both groups and were also compared in regards to demographics, nerve-sparing techniques, prostate size, cardiovascular risk profiles, and phosphodiesterase-5 inhibitor (PDE5i) uses. Logistic regression analyses were performed using age and other relevant clinicopathologic variables to predict PL recovery.

**Results:**

60.2% of the total study population regained their preoperative PL at 12 months. Average percent (length) differences from baseline were − 1.70% (− 0.25 cm) and − 16.42% (− 2.35 cm) in the CR and the IR groups, respectively (*p* < 0.001). Multivariate logistic regression demonstrated that younger age (OR 0.962; 95%CI 0.931–0.994; *p* = 0.019), high preoperative erectile function (EF) (OR 1.028; 95%CI 1.001–1.056; *p* = 0.046), and consistent PDE5i use (OR 1.998; 95%CI 1.166–3.425; *p* = 0.012) were independent predictors of CR. At 12-month follow up, PL difference for consistent PDE5iusers was statistically different from those who did not use PDE5i consistently (− 3.25%vs. -6.64%; *P* = 0.001).

**Conclusion:**

Age, preoperative EF, and consistent use of PDE5i were associated with complete recovery of baseline PL after RP. The therapeutic effect of PDE5i was most pronounced at 12-month visit, suggesting an added benefit with long-term use.

**Electronic supplementary material:**

The online version of this article (10.1186/s12894-018-0341-8) contains supplementary material, which is available to authorized users.

## Background

Urinary incontinence and erectile dysfunction (ED) are well-described postoperative complications associated with radical prostatectomy (RP). However, additional side effects of sexual nature have also been reported in the literature, such as orgasm-associated incontinence, altered orgasmic function, orgasm-associated pain, and penile length (PL) shortening [1].

In particular, PL shortening (PS) is a common occurrence and is experienced in 15–68% of patients after RP, with losses often greater than 1 cm in stretched penile length (SPL) [1, 2]. PS has been associated with compromised quality of life and self-esteem [3]. There are multiple mechanisms proposed for post-RP PS, including anatomic changes related to urethral shortening, neural damage and attendant ED, sympathetic overactivity associated with chronic contractions of the cavernous smooth muscle, and arterial insufficiency leading to hypoxia-induced smooth muscle apoptosis with subsequent fibrous tissue deposition [4]. However, the precise mechanism of PS remains unclear.

Additional evidence is necessary to resolve conflicting perspectives on the reversibility and natural history of post-RP PS. While some have argued that patients are unlikely to regain original PL and that permanent PS is inevitable at long-term follow-up [5, 6], others report that PL eventually returns to preoperative baseline at 9–48 months [7, 8]. The differences in recovery rate may be attributed to the heterogeneous study populations with varying demographic and clinical characteristics, and intraoperative techniques.

Several factors are suggested to be associated with better PL recovery including the presence of preoperative erectile function (EF) and a nerve-sparing (NS) RP [8, 9]. In addition, the use of phosphodiesterase-5 inhibitors (PDE5i) has been demonstrated to promote PL recovery, as PDE5i use has consistently shown to aid smooth muscle preservation after cavernosal nerve injury in animal models and in a randomized clinical trial [10–12]. However, whether anatomic alteration involving shortening of the urethra and pre-existing cardiovascular comorbidity contribute to PL recovery has not been adequately explored. Therefore, the aim of our study was to describe longitudinal patterns of PL recovery and evaluate potential factors predictive of complete PL recovery. We hypothesize that PDE5i use leads to long-term PL recovery compared to non-use.

## Methods

### Study cohort

Following Institutional Review Board (IRB) approval, a total of 602 patients who underwent robotic RP for localized prostate cancer (PCa) between May 2010 and December 2014 were queried after excluding subjects with Peyronie’s disease, previous penile surgery, or any other preexisting penile abnormalities. No individuals in our study cohort received vacuum, penile stretcher, or intracavernous injection therapies within the 12-month follow up period. Also, there were no cases of de-novo Peyronie’s diseases.

Demographic parameters [age, race, height, weight, cardiovascular (CV) risk factors, prostate-specific antigen (PSA), sexual health inventory for men (SHIM), history of transurethral resection of the prostate (TURP), and history of neo/adjuvant therapy], perioperative parameters [operative room (OR) time, estimated blood loss (EBL)], and postoperative parameters (pathologic stage, prostate weight, length, volume) were reviewed for each patient. Prostate volume was determined by measuring the overall size of the excised specimen in three dimensions, (i.e. apex-to-base, right-to-left, and anterior-to-posterior). Prostate length was measured in the vertical dimension from apex-to-base.

### Penile length

PL was measured in both flaccid and stretched states to the nearest 0.5 cm [13]. SPL was measured from pubic bone to coronal sulcus with the phallus maximally extended manually. SPL was utilized for all study analyses as a proxy for erected PL. All measurements of PL were performed by a single surgeon during a pre-operative visit before RP (defined as baseline PL), and then at post-operative visits at 7 days, and 3, 6, 9, and 12 months after RP. Although no repeat measurements were performed, each evaluation was performed blindly to prior measurements. The assessment took place in an exam room kept at a temperature ≥ 22 °C.

Comparisons were made between patients who had completely recovered (CR) their preoperative SPL within 1 year (CR: *N* = 397) and those who had incompletely recovered (IR: *N* = 131). Complete recovery was considered for men who achieved 100% of their preoperative PL in at least one of the five postoperative measurements without any specific follow-up requirement. Patients who did not achieve a full PL recovery were placed in IR group if they met a minimum of two separate follow-up visits, one of which included a 6 month-visit or later. Patients who failed to meet the follow-up requirement were excluded from the analysis (*N* = 74).

### Nerve sparing technique and pelvic lymph node dissection

The initial decision to preserve neurovascular bundle was made by thorough review of preoperative characteristics. However, the final decision to perform NS procedure was ultimately determined by intraoperative findings. In order to ensure that patients have optimal functional outcomes, efforts are made to perform NS techniques in the majority of patients, except for those with extensive high-volume disease. At our institution, two types of nerve sparing (NS) techniques are used: traditional interfascial approach (IF) and athermal intrafascial robotic (AIR) technique [14]. Hence, NS type was categorized as none, IF, or AIR. Additionally, patients were dichotomized by pelvic lymph node dissection (PLND) status.

### Cardiovascular risk factor

The presence of CV risk factors was determined based on past medical and surgical history along with the list of home medications queried from electronic medical records. Advanced CV risk was determined by the presence of coronary heart disease (CHD) or CHD-risk equivalents [15]. Men with a history significant for angina, myocardial infarction and/or surgical history of cardiac stent or coronary artery bypass graft were considered to have CHD-equivalent disease. Those with abdominal aortic aneurysm, peripheral vascular disease, and carotid artery disease were considered to have CHD-equivalents. Lastly, patients with regular use of clopidogrel were considered to have advanced CV risks as clopidogrel has been demonstrated to be a reliable surrogate marker for CV damage [16].

### Phosphodiesterase-5 inhibitor (PDE5i)

Post-operatively, patients were routinely recommended to take a daily PDE5i (Sildenafil, vardenafil, or tadalafil). Patients were placed on all three types of PDE5Is within the one-year period in the following sequential order: sildenafil 50 mg (Day 9–3 months), tadalafil 20 mg (4–6 months), sildenafil 100 mg (7–9 months), and vardenafil 20 mg (10–12 months). Using a three-point scale, PDE5i use was quantified as one of the following based on medication history as reported by patients: 1) almost always/always, 2) inconsistently, or 3) almost never/never.

### Erectile function rehabilitation

Patients who desired to engage in sexual intercourse were placed on our institutional erectile function rehabilitation protocol. The rehabilitation protocol utilized was a 12-month regimen that consisted of 2 parts: sexual stimulation and oral medication (Additional file 1: Table S1).

### Statistical analysis

PL recovery, in absolute length (cm) and percent (%) reduction from baseline, was compared at each postoperative visit using unpaired t-tests. Demographical and clinical variables were compared using chi-square (χ^2^) tests for categorical variables, and t-tests for continuous variables. Cohrane-Mantel-Haenszel chi-square test (χ^2^_CMH_) as well as Cochran-Armitage Trend Test were employed to measure the linear trend between PL recovery (CR vs. IR) and key demographic characteristics: age (< 50 vs. 50–60 vs. ≥ 60) and SHIM groups (1–7: low vs. 8–16: medium vs. ≥ 17: high).

Univariate and multivariate logistic regression analyses were performed using age and other variables to predict PL recovery. Logistic regression analysis was conducted with the inclusion of an interaction term to account for possible interaction between PDE5i use and preoperative SHIM. Longitudinal recovery patterns were analyzed according to PDE5i use and median age. All analyses were performed using SAS version 9.3 (SAS, Carry, NC). A two-sided *p* < 0.05 was considered significant.

## Results

**Fig. 1:**
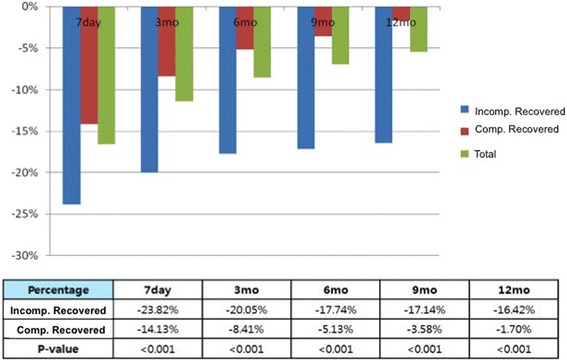
Comparing penile length recovery pattern between the completely recovered group and the incompletely recovered group, (**a**) Percent difference (preoperative-postoperative), % and (**b**) Length difference (preoperative-postoperative), cm

In the analysis of the PL recovery at various times, about half of the total study population regained their preoperative PL at 9 months in both flaccid and stretched lengths: 44.6% vs. 48.3%, respectively. Of those examined at 12 months, 59.4% and 60.2% of patients fully recovered in terms of flaccid and stretched measurements, respectively (Table 1). The percent reductions of PL in postoperative visits were significantly different between the CR and the IR group (all *p* < 0.002) (Fig. 1). Among those in the CR group, the mean initial PL reduction at 7-day follow-up was 14.13% (1.9 cm) and the final reduction at 12-month visit was 1.7% (0.25 cm) of the preoperative PL. The mean initial percent reduction in the IR group was 23.8% (3.38 cm), whereas the final reduction was 16.4% (2.36 cm) of the preoperative PL (Fig. 1a). The CR group showed steady recovery throughout the follow-up measurements, reaching less than 1 cm PL reduction (0.51 cm) by 9 months. The IR group showed a slower recovery, with mean reductions at 6, 9, and 12 months measured at − 2.51 cm, − 2.44 cm, and − 2.36 cm, respectively (Fig. 1b).

**Table 1:** Penile length recovery at various follow-up intervals

	Flaccid penile length		Stretched penile length	
	Analyzed, *N*	Recovered, *N* (%)	Analyzed, *N*	Recovered, *N* (%)
7 days	507	159 (31.4)	507	120 (23.7)
3 months	496	157 (31.7)	495	152 (30.7)
6 months	463	184 (39.7)	459	187 (40.7)
9 months	404	180 (44.6)	404	195 (48.3)
12 months	352	209 (59.4)	352	212 (60.2)

When comparing the 373 patients in the CR group with the 131 patients in the IR group, the CR group was younger (59.3 years vs. 62.0 years; *p* < 0.001) and had shorter preoperative penile measurements (8.38 cm vs. 8.93 cm; *p* < 0.0001). The CR group also had lower preoperative PSA (5.11 ng/ml vs. 5.50 ng/ml; *p* = 0.029) and had lower pathologic staging (≤T2: 78.9% vs. 68.5%; *p* = 0.017) (Additional file 2: Table S2). In terms of CV risk factors, the CR group had fewer patients with hypertension (40.3% vs. 50.8%; *p* = 0.047) and CAD (6.9% vs. 13.7%; *p* = 0.026).

While it appeared that a greater proportion of intraoperative NS was in the CR group versus the IR group (97.5% vs. 93.9%; *p* = 0.075), the anatomical parameters of the prostate including the weight (*p* = 0.56), volume (*p* = 0.93), and length (*p* = 0.46) were not associated with a full PL recovery. Regarding therapeutic management, a greater proportion of CR patients used PDE5i consistently when compared to the IR patients (31.7% vs. 16.0%; *p* = 0.002) (Additional file 2: Table S2).

The linear trends of PL recovery [CR within 3 months (‘Fast’ CR) vs. CR within 12 months (‘Slow’ CR) vs. IR] with respect to age (< 50 vs. 50–60 vs. > 60) and SHIM groups (1–7: low vs. 8–16: medium vs. > 17: high) were statistically significant (χ^2^_CMH_ = 9.0447and 12.83; *p* = 0.003 and 0.003, respectively). A decreasing trend of PL recovery with respect to age (< 50 vs. 50–60 vs. > 60) and increasing trend of recovery with respect to SHIM groups (1–7: low vs. 8–16: medium vs. > 17: high) were statistically significant (Cochran-Armitage Trend Test Z = − 3.74 and 3.85; *p* = 0.0002 and 0.0001, respectively).

Upon univariate logistic analysis, younger age (OR 0.944; 95%CI 0.916–0.973; *p* < 0.0001), higher preoperative SHIM (OR 1.049; 95%CI 1.023–1.075; *p* < 0.0001), and consistent use of PDE5i (OR 2.435; 95%CI 1.459–4.065; *p* = 0.001) were predictive of complete PL recovery. NS in either approach (AIR or IF), as opposed to a wide resection, showed a marginal association with complete PL recovery (OR 2.511; 95%CI 0.969–6.502; *P* = 0.058). In multivariate logistic analysis, however, only younger age (OR 0.962; 95%CI 0.931–0.994; *p* = 0.019), high preoperative SHIM (OR 1.028; 95%CI 1.001–1.056; *p* = 0.046), and consistent use of PDE5i (OR 1.998; 95%CI 1.166–3.425; *p* = 0.012) remained as the significant predictors of complete PL recovery when adjusting for NS status and other pertinent clinical parameters (Table 2). Evaluation of the effect of PDE5i use on PL stratified by the three SHIM categories revealed that the effect of PDE5i is significant at median SHIM level between 8 and 17 (OR 11.497; 95% CI 1.475–89.608; *p* = 0.020), but not at low SHIM level between 1 and 8 (OR 1.56; 95% CI 0.387–6.253; *p* = 0.53) or at high SHIM level between 17 and 25 (OR 1.47; 95% CI 0.777–2.773; *p* = 0.24) (Data not shown).

**Table 2:** Univariate and multivariate model predictive of complete penile length recovery

Variable	Univariate		Multivariate	
	Odds ratio (95% CI)	*P* value	Odds ratio (95%CI)	*P* value
Age	0.944 (0.916–0.973)	< 0.0001	0.962 (0.931–0.994)	0.019
PSA	0.977 (0.947–1.007)	0.13		
SHIM	1.049 (1.023–1.075)	< 0.0001	1.028 (1.001–1.056)	0.046
PDEi5				
None or Not consistent	Ref.		Ref.	
Always	2.435 (1.459–4.065)	0.001	1.998 (1.166–3.425)	0.012
NSS				
None	Ref.		Ref.	
AIR or Interfascial	2.511 (0.969–6.502)	0.058	1.676 (0.602–4.665)	0.32

Abbreviations: *PSA* Prostate-specific antigen, *SHIM* Sexual health index of male, *PDEi5* Phosphodiesterase inhibitor-5, *NSS* Nerve-sparing surgery, *AIR* Athermal intrafacial robotic technique

**Fig. 2:**
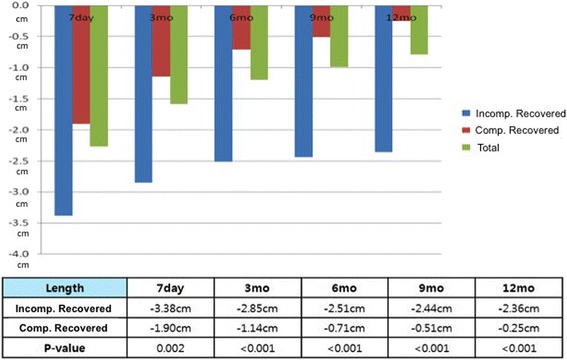
Penile length recovery pattern according to PDE-5 inhibitor use

When stratified by PDE5i use, patients with consistent PDE5i use had improved recovery patterns at the 12-month visit compared to those who did not take PDE5i consistently (− 3.25% vs. -6.64%; *p* < 0.001) (Fig. 2). Alternatively, the study population was divided based on whether patients had attained median study age (≥ 60 vs. < 60). These two age groups showed comparable recovery patterns (Data not shown).

## Discussion

Despite concerns that post-RP PS may be irreversible [5, 6], our results demonstrate that 59.4% and 60.2% of patients returned to their baseline stretched and flaccid PL, respectively, suggesting that PS is not a permanent consequence. Additionally, our study demonstrated that younger age, high preoperative erectile function, and consistent PDE5i use were independent predictors of complete PL recovery. It appears that these therapeutic effects of PDE5i were only appreciated for long-term users, and in individuals with medium preoperative SHIM scores. Neither prostate anatomy nor metabolic risk factors were significant predictors of complete PL recovery.

Older patients were less likely to reach complete PL recovery when controlled for other variables. This finding agrees with the results of a recent report that analyzed postoperative EF and continence, confirming the correlation between senility and delayed recovery from surgical trauma [17]. Among other variables associated with complete recovery was a high baseline preoperative EF, which has also been previously identified as a protective factor in PL recovery [8, 9]. Similarly, our multivariate logistic regression model revealed that high preoperative SHIM, a marker for intact erectile function, was an independent predictor of complete PL recovery.

NS techniques have frequently been reported as an independent predictor of reduced PL loss [5]. One study demonstrated that patients undergoing NS RP had no changes in penile measurements at 6-months postoperative visit when compared to preoperative baseline [9]. Our univariate logistic regression showed that both types of NS RP approach were marginally predictive of complete PL recovery (OR 2.511; 95%CI 0.969–6.502; *p* = 0.058). The lack of association at the multivariate level could be related to the distribution of our study cohort where 96.6% of men were operated on using a NS technique - either IF or AIR. No difference was appreciated in PL recovery between these two types of NS techniques (*p* = 0.24) although AIR was found to be a superior NS technique when compared to IF in terms of preserving sexual function in our earlier report [14].

Prostate anatomic factors have been implicated in the pathophysiology of PL shortening because a part of prostatic urethra will be resected during surgery [13]. However, recent studies demonstrated that prostate size and weight were not correlated with PS, suggesting that the length of prostatic urethra is fixed at the urogenital diaphragm [2, 5]. However, no study has investigated the effects of post-surgical anatomic alteration using more than two parameters. The incorporation of comprehensive anatomic parameters in our study has shown that prostate length (4.4 cm vs. 4.3 cm; *p* = 0.93), volume (63.7 ml vs. 64.1 ml; *p* = 0.46), and weight (49.1 g vs. 50.7 g; *p* = 0.40) were not significantly different between the CR and the IR groups, demonstrating that prostate size itself does not affect PL recovery.

We also investigated the effects of metabolic derangement and CV risk factors on PL recovery. In our findings, the CR group had a smaller proportion of men affected by hypertension and CAD when compared to the IR group (40.3% vs. 50.8%; *p* = 0.047 and 6.9% vs. 13.7%; *p* = 0.026, respectively), but the associations with CV factors did not remain significant in logistic regression analyses, suggesting that they might be an age-related phenomenon. Similarly, the proportion of diabetic patients who reached a complete recovery were similar in both the CR and the IR groups (13.0% vs. 12.1%; *p* = 0.877).

The literature suggests that patients can improve PL recovery by consistently taking PDE5i. Recently, Brock et al. reported on 423 patients who were randomized to receive 1) 5-mg tadalafil once daily (OaD), 2) 20-mg tadalafil on-demand (“pro renata”, PRN), or 3) placebo [12]. The authors found that at the end of 9 months, PS was significantly less for patients treated with tadalafil OaD than those treated with placebo, with a least-squares mean difference in stretched PL change from preoperative PL of 4.1 mm (95% CI, 0.4–7.8; *P* = 0.032). No significant difference in PL change was observed between tadalafil PRN and placebo.

Our results are in line with previous studies and support the routine use of PDE5i after RP. Our multivariate logistic analysis demonstrated that consistent use of PDE5i was predictive of PL recovery (OR 1.998; 95%CI 1.166–3.425; *p* = 0.012). Moreover, when the study population was analyzed according to PDE5i use, the difference in penile recovery was only significant at 12-month postoperative visit (Fig. 2).

Our study is unique in that it not only attempted to describe longitudinal patterns of PL recovery between the CR and the IR groups, but also analyzed comprehensive factors associated with complete recovery within a one-year follow-up. Our study has confirmed many previous findings, and also has tested claims that were largely speculative. Importantly, our study further supports the long-term efficacy of consistent PDE5i use for PL recovery. This benefit of PDE5i in this patient population was first proved in the recent clinical trial that demonstrated that tadalafil has therapeutic potential beyond its conventional indication. To add to this, our study has found that the PL protective effect is not just unique to patients using tadalafil, but also to patients using sildenafil and vardenafil, thereby better representing the current clinical practice where different types of PDE5i are utilized in a heterogeneous patient population. For example, our study population is not limited to patients with Gleason 6 disease and PSA < 10 ng/mL, two of the inclusion criteria listed for the clinical trial. To our knowledge, our study is also the first to describe the temporal patterns of PDE5i efficacy and identify SHIM scores that would benefit from PDE5i use for PL recovery, defining a target population for intervention. The results of our study are also important to patients, as PL shortening has been consistently associated with a decreased quality of life, including reduced self-esteem, interference with close relationships, and ultimately treatment regret [18, 19]. The potential of PDE5is to potentially improve PL recovery, and consequently quality of life, is a concept that many patients may wish to embrace.

Nonetheless, our study is limited by several weaknesses. First, limited by its retrospective design with inherent selection bias, our study necessitates a randomized clinical trial for a higher level of evidence and confirmation of our results. Second, it is possible that the frequency of sexual intercourse and patient/partner satisfaction are correlated with the rate of PL recovery, but this information was unattainable in our study. Third, a longer follow-up beyond 1 year would be more useful to understand patterns of PL recovery in patients who demonstrated slower PL recovery. Fourth, measurement errors were inevitable. Although inter-observer bias was eliminated as a single surgeon evaluated patients throughout the study period, intra-observer variability may not have been well-controlled for as repeat measurements were not made for each visit. Fifth, the design of our penile habitation protocol also makes a direct comparison of the three PDE5I agents not possible because our PDE5i assignment regimen entails that a patient would take all three types in an assigned, sequential order. But it also provided an opportunity for patients to explore because it is reported that up to 40% patients are kept from best drug of choice if they only try one type of PDE5Is [20]. Sixth, patients who received adjuvant or salvage radiation therapies were not excluded from the study, but the relationship between penile length recovery and radiation therapy was not statistically significant in our study cohort (Additional file 2: Table S2). The cost of PDE5is is a potential financial limitation and may be prohibitive to some patients, though a generic version of sildenafil is now currently available. Lastly, our penile measurement did not include penile girth, and the standardized approximation of erect PL from SPL could be a source of systemic bias. However, it is not feasible to perform direct measurement of erect PL, and SPL is considered an accepted alternative [13].

## Conclusion

Our study demonstrated that more than half of post RP patients regain their preoperative PL by one-year follow up. There is evidence that a long-term use of PDE5i may aide PL recovery, particularly in individuals with medium SHIM scores. In agreement with current literature, age and preoperative erectile function were also associated with complete recovery of baseline PL.

## Additional files

Additional file 1: **Table S1.** Erectile Function Rehabilitation Protocol. (DOC 29 kb)

Additional file 2: **Table S2.** Baseline study cohort characteristics. (DOC 67 kb)

## Abbreviations

χ^2^: Chi-square
AIR: Athermal intrafascial robotic
CAD: Coronary artery disease
CHD: Coronary heart disease
CI: Confidence interval
CR: Complete recovery
CV: Cardiovascular
EBL: Estimated blood loss
ED: Erectile dysfunction
EF: Erectile function
IF: Interfascial approach
IR: Incomplete recovery
NS: Nerve-sparing
OR: Operative room (OR)
PCa: Prostate cancer
PDE5i: Phosphodiesterase-5 inhibitor
PL: Penile length
PLND: Pelvic lymph node dissection
PRN: Pro re nata
PS: Penile length shortening
PSA: Prostate-specific antigen
RP: Radical prostatectomy
SHIM: Sexual health inventory for men
SPL: Stretched penile length
TURP: Transurethral resection of the prostate
