# Supplementary material for: Longitudinal recovery patterns of penile length and the underexplored benefit of long-term phosphodiesterase-5 inhibitor use after radical prostatectomy
Source: BMC Urol. 2018 May 9;18:37. doi: 10.1186/s12894-018-0341-8 (PMC5941788; doi:10.1186/s12894-018-0341-8)
Supplement: Supplementary file 1 — Table S1. Erectile Function Rehabilitation Protocol. (DOC 29 kb) [file 12894_2018_341_MOESM1_ESM.doc]

**Table S1. Erectile Function Rehabilitation Protocol**

|  | ***Day 1*** | ***Day 8*** | ***Day 9 –***  ***3 months*** | ***Months 4—6*** | ***Months 7—9*** | ***Months 10—12*** |
| --- | --- | --- | --- | --- | --- | --- |
| ***Procedure*** | RP | Foley catheter removal |  |  |  |  |
| ***Drug*** | - | - | Sildenafil | Tadalafil | Sildenafil | Vardenafil |
| ***Dose*** |  |  | 50 mg | 20 mg | 100 mg | 20 mg |
| ***Frequency*** |  |  | EOD | EOD for 1 wk  DFW for 2 wks  Repeat cycle  (1 week on, 2 weeks off)  for 3 months | EOD for 1 wk  DFW for 2 wks  Repeat cycle  (1 week on, 2 weeks off)  for 3 months | EOD for 1 wk  DFW for 2 wks  Repeat cycle  (1 week on, 2 weeks off)  for 3 months |
| ***Time*** |  |  | Before bed | In the morning | 3 hrs after dinner | 3 hrs after lunch |

Abbreviations: RP, radical prostatectomy; EOD, every other day; end DFW, drug free week
