# Supplementary material for: Longitudinal recovery patterns of penile length and the underexplored benefit of long-term phosphodiesterase-5 inhibitor use after radical prostatectomy
Source: BMC Urol. 2018 May 9;18:37. doi: 10.1186/s12894-018-0341-8 (PMC5941788; doi:10.1186/s12894-018-0341-8)
Supplement: Supplementary file 2 — Table S2. Baseline study cohort characteristics. (DOC 67 kb) [file 12894_2018_341_MOESM2_ESM.doc]

**Table S**2. Baseline study cohort characteristics

|  |  | **Stretched penile length recovery** | | |
| --- | --- | --- | --- | --- |
|  |  | **IR (N=131)** | **CR (N=397)** | **P- value** |
| **Age, *years*, mean (SD)** |  | 62.0 (6.75) | 59.3 (6.90) | <0.0001 |
| **Race, N (%)** | **White** | 111 (84.7) | 315 (79.3) | 0.253 |
|  | **AA** | 12 (9.2) | 58 (14.9) |  |
|  | **Others** | 8 (6.1) | 22 (5.9) |  |
| **BMI, *kg/m2*, mean (SD)** |  | 31.3 (11.28) | 30.9 (6.38) | 0.634 |
| **Preoperative PSA,**  ***ng/ml*, median (SD)** |  | 5.50 (30.85) | 5.11 (5.56) | 0.029 |
| **Hypertension, N (%)** | **No** | 61 (49.2) | 225 (59.7) | 0.047 |
|  | **Yes** | 63 (50.8) | 152 (40.3) |  |
| **Diabetes mellitus, N (%)** | **No** | 109 (87.9) | 328 (87.0) | 0.877 |
|  | **Yes** | 15 (12.1) | 49 (13.0) |  |
| **Dyslipidemia, N (%)** | **No** | 70 (56.5) | 244 (64.7) | 0.109 |
|  | **Yes** | 54 (43.5) | 133 (35.3) |  |
| **CAD, N (%)** | **No** | 107 (86.3) | 351 (93.1) | 0.026 |
|  | **Yes** | 17 (13.7) | 26 (6.9) |  |
| **TURP, N (%)** | **No** | 127 (96.9) | 390 (98.2) | 0.773 |
|  | **Yes** | 4 (3.1) | 7 (1.8) |  |
| **Adjuvant/salvage therapy, N (%)** | **No** | 122 (93.1) | 378 (95.2) | 0.371 |
|  | **Yes** | 9 (6.9) | 19 (4.8) |  |
| **Preop Penile measurement,**  ***cm*, mean (SD)** | **Flaccid** | 8.93 (1.37) | 8.38 (1.38) | <0.0001 |
|  | **Stretched** | 14.2 (1.59) | 13.5 (1.80) | 0.001 |
| **Preoperative SHIM, mean (SD)** |  | 14.2 (8.22) | 17.3 (7.70) | <0.0001 |
| **Preoperative SHIM, N (%)** | **≥ 17** | 57 (45.2) | 248 (63.6) | 0.001 |
|  | **≥8 and < 17** | 33 (26.2) | 80 (20.5) |  |
|  | **< 8** | 36 (28.6) | 62 (15.9) |  |
| **PDE5i use, N (%)** | **Always** | 21 (16.0) | 126 (31.7) | 0.002 |
|  | **Not consistent** | 77 (58.8) | 181 (45.6) |  |
|  | **None** | 33 (25.2) | 90 (22.7) |  |
| **Estimated blood loss,**  ***ml*, median (SD)** |  | 200 (198.8) | 200 (185.1) | 0.923 |
| **Operative room times,**  ***minute*, mean (SD)** |  | 202.2 (43.9) | 202.5 (65.7) | 0.960 |
| **Nerve sparing, N (%)** | **No** | 8 (6.1) | 10 (2.5) | 0.075 |
|  | **Yes (AIR)** | 63 (48.1) | 221 (55.8) |  |
|  | **Yes (Interfascial)** | 60 (45.8) | 165 (41.7) |  |
| **BL PLND, N (%)** | **No** | 92 (70.2) | 302 (76.1) | 0.203 |
|  | **Yes** | 39 (29.8) | 95 (23.9) |  |
| **Margin status, N (%)** | **No** | 90 (68.7) | 292 (73.6) | 0.311 |
|  | **Yes** | 41 (31.3) | 105 (26.4) |  |
| **Prostate weight, *gram*, mean (SD)** |  | 50.5 (19.0) | 49.4 (18.0) | 0.561 |
| **Prostate volume, *ml*, mean (SD)** |  | 64.1 (36.5) | 63.7 (33.9) | 0.929 |
| **Prostate length, *cm*, mean (SD)** |  | 4.3 (0.97) | 4.4 (0.87) | 0.461 |
| **Pathologic stating, N (%)** | **≤T2** | 89 (68.5) | 307 (78.9) | 0.017 |
|  | **≥T3** | 41 (31.5) | 82 (21.1) |  |

Abbreviations: IR, Incomplete recovery; CR, Complete recovery; SD, Standard deviation; BMI, Body mass index; CAD, Coronary artery disease; TURP, Transurethral resection of the prostate; SHIM, sexual health index of male; PDE5i, phosphodiesterase-5 inhibitor; AIR, Atermal intrafascial robotic technique; BL PLND; Bilateral pelvic lymph node dissection
